# Long Non-Coding RNAs H19 and HOTAIR Implicated in Intervertebral Disc Degeneration

**DOI:** 10.3389/fgene.2022.843599

**Published:** 2022-03-03

**Authors:** Zhun Wang, Jingwei Zhang, Wei Zheng, Yongjin He

**Affiliations:** Department of Pain, Tianjin First Central Hospital, Tianjin, China

**Keywords:** H19, HOTAIR, differentially expressed genes, JAK–STAT pathway, intervertebral disc degeneration

## Abstract

**Objective:** Intervertebral disc degeneration (IDD) is the major cause of low back pain. We aimed to identify the key genes for IDD pathogenesis.

**Methods:** An integrated analysis of microarray datasets of IDD archived in public Gene Expression Omnibus was performed. Bioinformatics analyses including identification of differentially expressed mRNAs/microRNAs/long non-coding RNAs (DEMs/DEMis/DELs), pathway enrichment, and competitive endogenous RNA (ceRNA) network construction were performed to give insights into the potential functions of differentially expressed genes (DEGs, including DEMs, DEMis, and DELs). The diagnostic value of DEMis in distinguishing IDD from normal controls was evaluated through receiver operating characteristic (ROC) analysis.

**Results:** DEGs were identified in IDD, including H19 and HOTAIR. In the DEMis–DEMs network of IDD, miR-1291, miR-4270, and miR-320b had high connectivity with targeted DEMs. Cell death biological processes and the JAK–STAT pathway were significantly enriched from targeted DEMs. The area under the curve (AUC) of 10 DEMs including miR-1273e, miR-623, miR-518b, and miR-1291 in ROC analysis was more than 0.8, which indicated that those 10 DEMs had diagnostic value in distinguishing IDD from normal individuals.

**Conclusions:** DELs H19 and HOTAIR were related to IDD pathogenesis. Cell death biological processes and the JAK–STAT pathway might play key roles in IDD development.

## Introduction

Spinal degenerative disease is a major health problem with a social burden worldwide. Intervertebral disc degeneration (IDD) is a major cause of back, neck, and radicular pain, which is characterized by the loss of nucleus pulposus cell (Waddell, 1996; Andersson, 1999; Ma et al., 2016).

The intervertebral disc comprises an outer circumferential annulus fibrosus (AF) and an inner nucleus pulposus (NP), bordered by two cartilaginous endplates (Johnson et al., 2015). It is reported that IDD is linked to various pro-inflammatory cytokines. Recently, a series of articles displayed non-coding RNAs, such as circular RNAs (circRNAs), long non-coding RNAs (lncRNAs), and microRNAs (small endogenous RNAs that posttranscriptionally regulate gene expression), are involved in the initiation and progression of IDD. lncRNA HCG18 suppresses the growth of NP cells and promotes the IDD development through the miR-146a-5p/TRAF6/NFκB axis (Xi et al., 2017). The overexpression of miR-146a could promote IDD through the TRAF/NF-κB pathway (Lv et al., 2017). Decreased miR-155 contributes to the up-regulation of MMP-16 *in vivo*, which degrades aggrecan and collagen type II, leading to the dehydration and degeneration of discs (Zhang et al., 2017).

Although great progress has been made in the mechanism research of IDD, the mechanisms of initiation and development in IDD remain elusive. In the present work, we performed an integrated analysis of public microarray datasets of IDD to elaborate IDD pathogenesis and identify non-coding RNAs to distinguish IDD patients from healthy controls for potential clinical management.

## Materials and Methods

### Microarray Datasets

Gene Expression Omnibus (GEO) (www.ncbi.nlm.nih.gov/geo/) is an international public database that archives high-throughput sequencing gene expression data. In order to explore the different expression profiling in IDD, we searched datasets from the GEO database with the keywords “intervertebral disc degeneration” AND “*Homo sapiens*” AND “gse”. Expression data generated from the human annulus disc tissue of IDD patients and healthy controls were incorporated into our work. Four mRNA expression datasets (including GSE23130, GSE15227, GSE17077, and GSE70362) and 3 miRNA expression datasets (GSE116726, GSE63492, and GSE45856) were included. The basic information of datasets is shown in Table 1. This study has been approved by the Ethics Committee of Tianjin First Central Hospital.

**TABLE 1:** Detail information of microarray datasets.

Dataset	HC	IDD	Platform	Year	Country	Author	PMID
**mRNA expression profiling (52 HC vs. 31 IDD)**							
GSE23130	17	8	GPL1352 [U133_X3P] Affymetrix Human X3P Array	2011	United States	Helen Gruber	-
GSE15227	12	3	GPL1352 [U133_X3P] Affymetrix Human X3P Array	2009	United States	Helen Gruber	19535298
GSE17077	9	10	GPL1352 [U133_X3P] Affymetrix Human X3P Array	2009	United States	Helen Gruber	20109216
GSE70362	14	10	GPL17810 [HG-U133_Plus_2] Affymetrix Human Genome U133 Plus 2.0 Array	2015	Ireland	Peadar O'Gaora	26489762
**miRNA expression profiling (11 HC vs. 11 IDD)**							
GSE116726	3	3	GPL20712 Agilent-070156 Human miRNA [miRNA version]	2018	China	Jian Chen	30487517
GSE63492	5	5	GPL19449 Exiqon miRCURY LNA microRNA Array, 7th generation REV- hsa, mmu, and rno (miRBase v18.0)	2016	China	Hai-Qiang Wang	26484230
GSE45856	3	3	GPL11434 miRCURY LNA microRNA Array, 6th generation- hsa, mmu, and rno	2014	China	BO Zhao	24173697

### Differentially Expressed mRNAs/miRNAs/circRNA/lncRNA in IDD

In order to minimize the heterogeneity among different datasets, raw data were performed for log2 transformation and normalization, and then, the metaMA package was used to combine data from multiple datasets. Individual *p* values were calculated, and false discovery rate (FDR) was obtained by using the Benjamini–Hochberg method. Differentially expressed mRNAs (DEMs), miRNAs (DEMis), circRNAs (DECs), and lncRNAs (DELs) were investigated in IDD. In our work, mRNAs with *p* value < 0.05, miRNAs with FDR<0.01, circRNAs with FDR<0.01, and lncRNAs with FDR<0.01 were considered as DEMs, DEMis, DECs, and DELs, respectively.

### miRNA Regulatory Network Construction

The DEM-associated target genes were predicted using the miRwalk3 (http://mirwalk.umm.uni-heidelberg.de/), which stores predicted data obtained with a machine learning algorithm including experimentally verified miRNA–target interactions. The target genes were then overlapped with the DEMs, and the negative interaction pairs between DEMis and DEMs (according to their expression levels) were used to construct the DEMi–DEM network using Cytoscape software (version 3.6.1; www.cytoscape.org).

### CeRNA Regulatory Network Construction

The starBase database (version 2.0; starbase.sysu.edu.cn/index.php) (Cardenas-Gonzalez et al., 2017) was used to screen the interactions between DELs and DEMis, which were then integrated with the miRNA–mRNA interactions to establish the DEL–DEMi–DEM ceRNA network using Cytoscape software (version 3.6.1; www.cytoscape.org). Human sequences of DECs and DEMis were downloaded from the circBase (www.circbase.org) (Li et al., 2020) and miRBase (version 21; www.mirbase.org) (Chen et al., 2019) databases, respectively. miRanda (cbio.mskcc.org/miRNA2003/miranda.html) (Ren et al., 2020) was used to predict the interactions between DECs and DEMis. The interaction pairs between DECs and DEMs were then integrated with the DEMi–DEM interactions to establish the DEC–DEMi–DEM ceRNA network using Cytoscape software (version 3.6.1; www.cytoscape.org). The overlapped DEMi–DEM in the aforementioned two ceRNA networks was also selected to construct the lncRNA/circRNA–miRNA–mRNA network.

### ROC Curve Analysis

In order to explore the diagnostic value of DEMis in IDD, the pROC package in R language curves was used to depict the receiver operating characteristic (ROC), and the area under the curve (AUC) was calculated. The DEMis with AUC≥0.8 were considered as having the performance of distinguishing IDD patients from healthy controls. The diagnostic value of DEMis in GSE116726, GSE63492, and GSE45856 datasets was investigated in the present study.

### Enrichment of Biological Function

The biological functions of DEMs in IDD were predicted by both Kyoto Encyclopedia of Genes and Genomes (KEGG) and Gene Ontology (GO) function and pathway through online software Genecodis3 (http://genecodis.cnb.csic.es) as a non-redundant and modular enrichment analysis tool for functional genomics (Tabas-Madrid et al., 2012). The enriched KEGG pathway with FDR <0.05 was the significant enrichment term.

### Validation of the Expression Level of DECs, DELs, and DEMs

The expression levels of DEC, DEL, and DEM candidates were explored in external datasets, including GSE124272, GSE150408, and GSE153761. Both GSE124272 (8 IDD patients and 8 healthy individuals) and GSE150408 (17 IDD patients and 17 healthy individuals) store transcriptomic profiling data of the whole blood of patients with IDD and healthy individuals. GSE153761 (3 IDD patients and 3 healthy individuals) stores transcriptomic profiling data of the cervical cartilage endplate of patients with IDD and healthy individuals.

### Statistical Analysis

The statistical significance between groups was assessed by unpaired Student’s t-test. *p* < 0.05 was statistically significant. * indicates *p* < 0.05; ** indicates *p* < 0.01, and *** indicates *p* < 0.001.

## Results

### Different Expression Analysis

**FIGURE 1:**
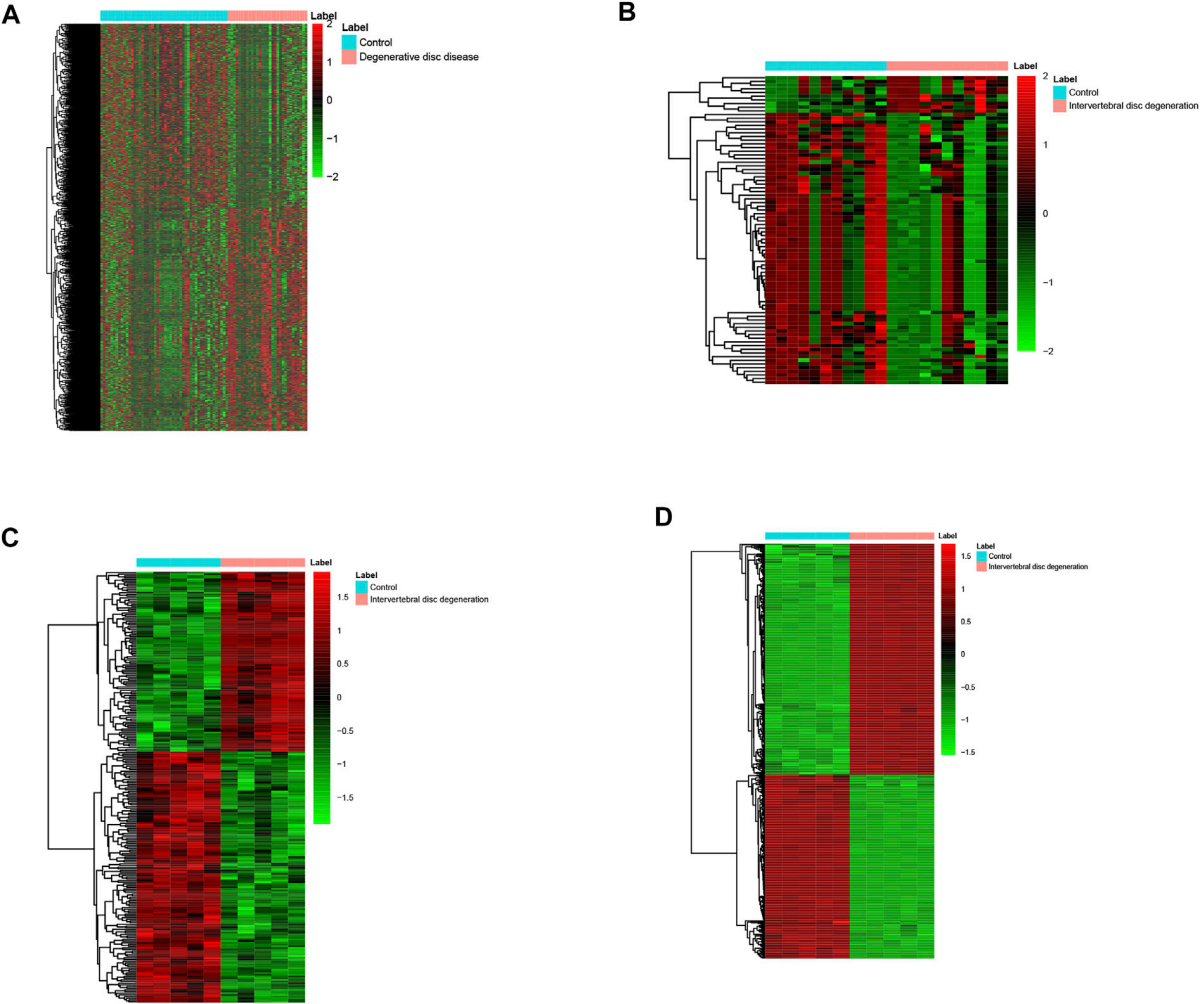
Heatmap of DECs **(A)**, DELs **(B)**, DEMis **(C)**, and DEMs **(D)** in IDD and healthy individuals. DECs: differentially expressed circular RNAs; DEL: differentially expressed long non-coding RNAs; DEMi: differentially expressed microRNAs: DEMs: differentially expressed mRNAs; and IDD: intervertebral disc degeneration.

A total of four mRNA expression datasets including 52 healthy controls (HCs) and 31 IDD patients were used to identify the DEMs in IDD. A total of 1995 DEMs including 907 down-regulated and 1,078 up-regulated DEMs were identified in IDD vs. HC. Three miRNA expression datasets including 11 HCs and 11 IDD patients were used to identify the DEMis in IDD. Eighty-four DEMis including 10 up-regulated DEMis and 74 down-regulated DEMis were identified in IDD compared with HCs. A total of 256 DELs including 107 up-regulated and 149 down-regulated DELs were identified in IDD patients. In addition, 589 DECs including 328 up-regulated and 261 down-regulated DECs were identified in IDD patients. The hierarchical cluster heatmaps indicated that these DECs (Figure 1A), DELs (Figure 1B), DEMis (Figure 1C), and DEMs (Figure 1D) could distinguish IDD from control samples.

### miRNAs–Target Genes Network

**FIGURE 2:**
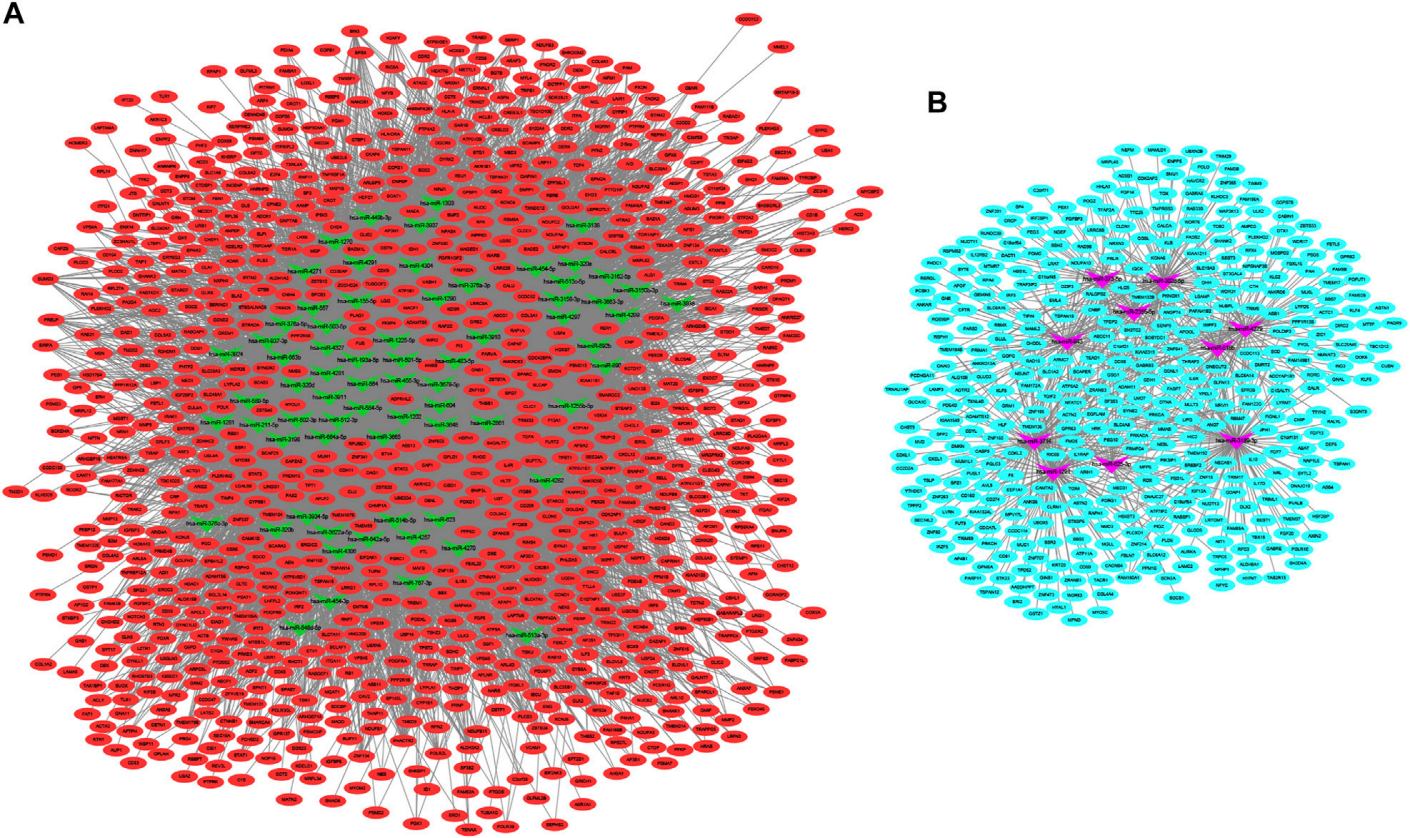
DEMis-targeted DEMs network in IDD. **(A)** Network among down-regulated DEMis and up-regulated DEMs; **(B)** network among up-regulated DEMis and down-regulated DEMs; green and purple node indicated down-regulated DEMis and up-regulated DEMis, respectively, and red and turquoise node indicated up-regulated DEMs and down-regulated DEMs, respectively. DEMis: differentially expressed microRNAs; DEMs: differentially expressed mRNAs; and IDD: intervertebral disc degeneration.

We constructed the interaction network between miRNAs and target genes in IDD based on the identified miRNA–target gene interaction pairs of reverse association using Cytoscape software. As shown Figure 2, 8,673 miRNA–target gene pairs of reverse correlation between 80 DEMis and 1,268 DEMs were identified in IDD, which included 7,906 pairs between 70 down-regulated DEMis and 885 up-regulated DEMs (Figure 2A) and 767 pairs between 10 up-regulated DEMis and 383 down-regulated DEMs (Figure 2B). In up-regulated DEMis pairs, miR-3189-3p, miR-3714, miR-1291, and miR-302c-5p had the high connectivity with DEMs, which interacted with 126, 112, 97, and 93 DEMs. In the down-regulated DEMis pairs, miR-4270, miR-3162-5p, miR-320b, miR-3138, miR-3198, miR-3679-5p, miR-3622a-5p, and miR-4257 interacted with more than 200 DEMs, respectively.

**FIGURE 3:**
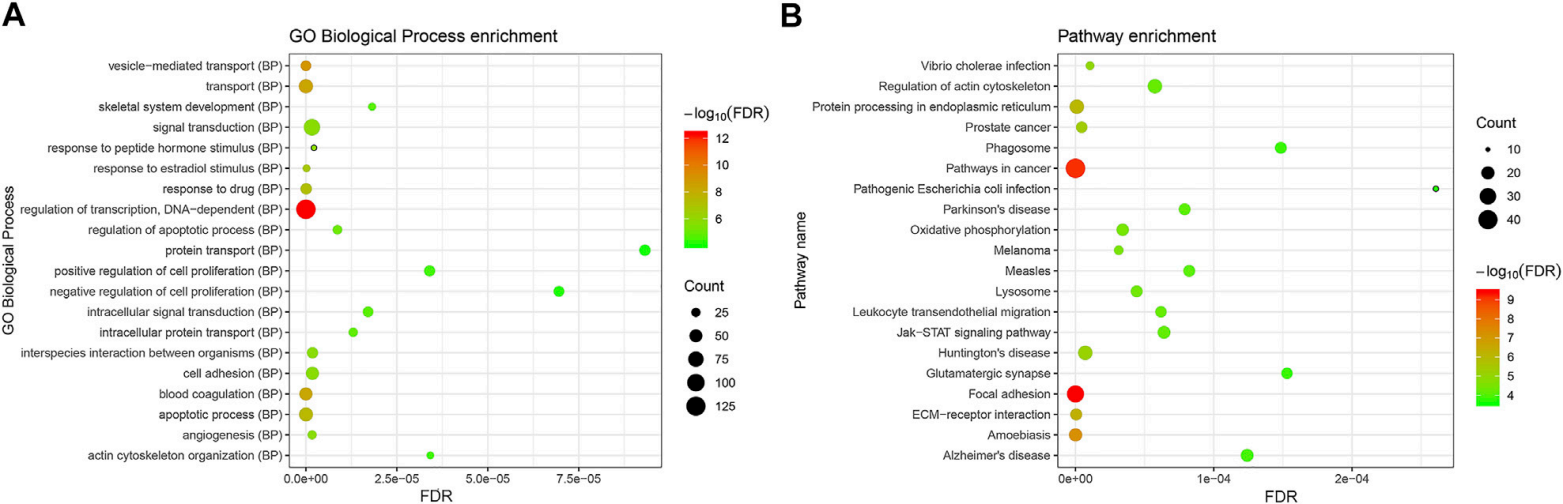
The enriched biological functions and pathways of the DEMs in the DEMis-targeted DEMs network in IDD. **(A)** The enriched biological process of targeted DEMs; **(B)** the enriched KEGG pathway of targeted DEMs. DEMs: differentially expressed mRNAs; IDD: intervertebral disc degeneration; and KEGG: Kyoto Encyclopedia of Genes and Genomes.

The underlying functions of the DEMs in the miRNA–mRNA network were also analyzed using the Genecodis database as described in *Materials and Methods*. The biological process GO annotation and KEGG pathway of target genes of DEMis with FDR <0.05 were considered as significant enrichment terms. As Figure 3A shows, skeletal system development and cell death biological processes including the apoptotic process and regulation of apoptotic process with FDR <0.05 were significantly enriched. The JAK–STAT signaling pathway, ECM-receptor interaction, Parkinson’s disease, and Alzheimer’s disease with FDR <0.05 were also significantly enriched (Figure 3B).

### ceRNA Network

**FIGURE 4:**
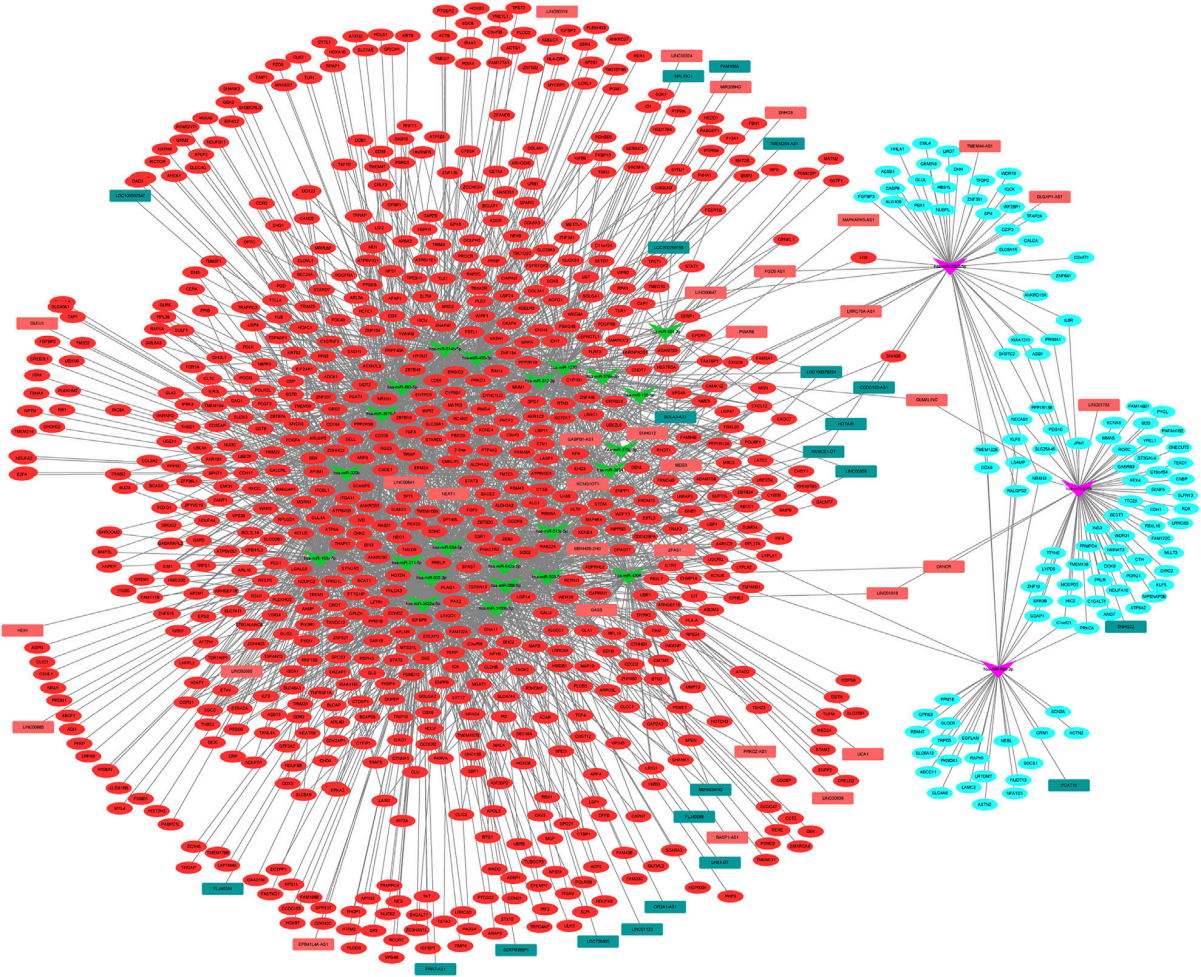
lncRNA–miRNA–mRNA ceRNA network among DELs, DEMis, and DEMs in IDD. DEL: differentially expressed long non-coding RNAs; DEMi: differentially expressed microRNAs; DEMs: differentially expressed mRNAs; IDD: intervertebral disc degeneration; and ceRNA: competitive endogenous RNA.

Using the starBase database, 26 DEMis were predicted to regulate 55 DELs; this was used to establish the lncRNA–miRNA–mRNA ceRNA network via integration with the miRNA–mRNA network (Figure 4). This network comprised 933 nodes (26 DEMis, 55 DELs, and 852 DEMs) and 2,801 interactions. Notably, down-regulated HOTAIR may function as a ceRNA to suppress the inhibitory effects of hsa-miR-454-3p on CRNKL1 and PDGFRB and hsa-miR-642a-5p on MMP13, MAP4K4, and PIK3R1, thus leading to their up-regulated expression. Similarly, up-regulated H19 may regulate the targeted effects of hsa-miR-454-3p on CRNKL1 and PDGFRB, as well as hsa-miR-2355-5p on PBX1 and TFDP2. Functional analysis of genes in the lncRNA-related ceRNA network revealed that they were significantly enriched in leukocyte transendothelial migration, focal adhesion, and ECM-receptor interaction.

**FIGURE 5:**
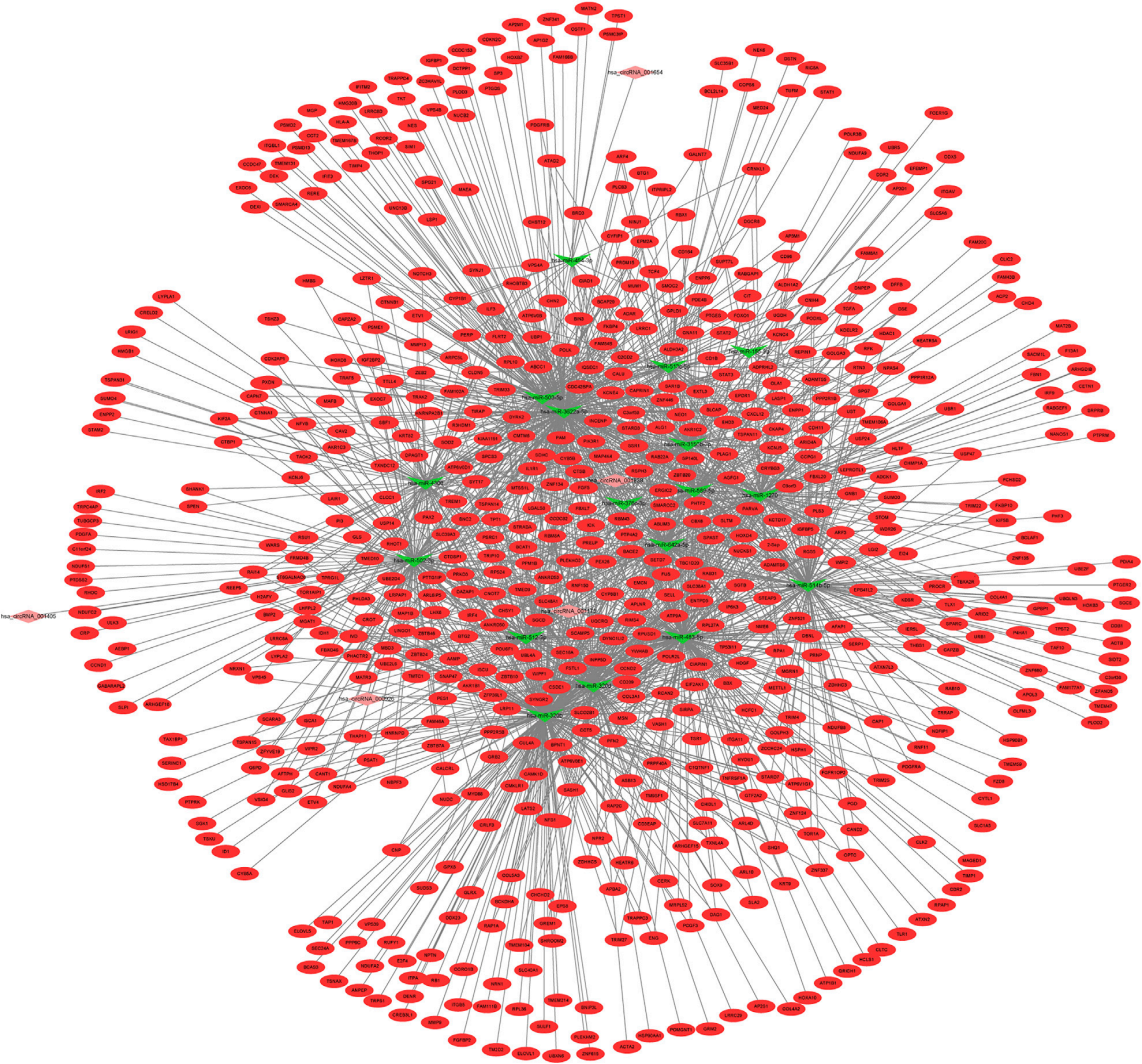
circRNA–lncRNA–mRNA ceRNA network among DECs, DEMLs, and DEMs in IDD. DEC: differentially expressed circular RNAs; DELs: differentially expressed long non-coding RNAs; DEMs: differentially expressed mRNAs; IDD: intervertebral disc degeneration; and ceRNA: competitive endogenous RNA.

Using the miRanda database, 5 DECs were predicted to regulate 17 DEMis; this information was used to establish the circRNA–lncRNA–mRNA ceRNA network via integration with the miRNA–mRNA network (Figure 5). Notably, up-regulated hsa_circRNA_001838 may function as a ceRNA to suppress the inhibitory effects of hsa-miR-4306 on FAM46A, VASH1, and CMTM6, thus resulting in their up-regulated expression. Functional analysis of genes in the lncRNA-related ceRNA network revealed that they were significantly enriched in leukocyte transendothelial migration, focal adhesion, and ECM–receptor interaction.

### ROC Analysis of DEMis

**FIGURE 6:**
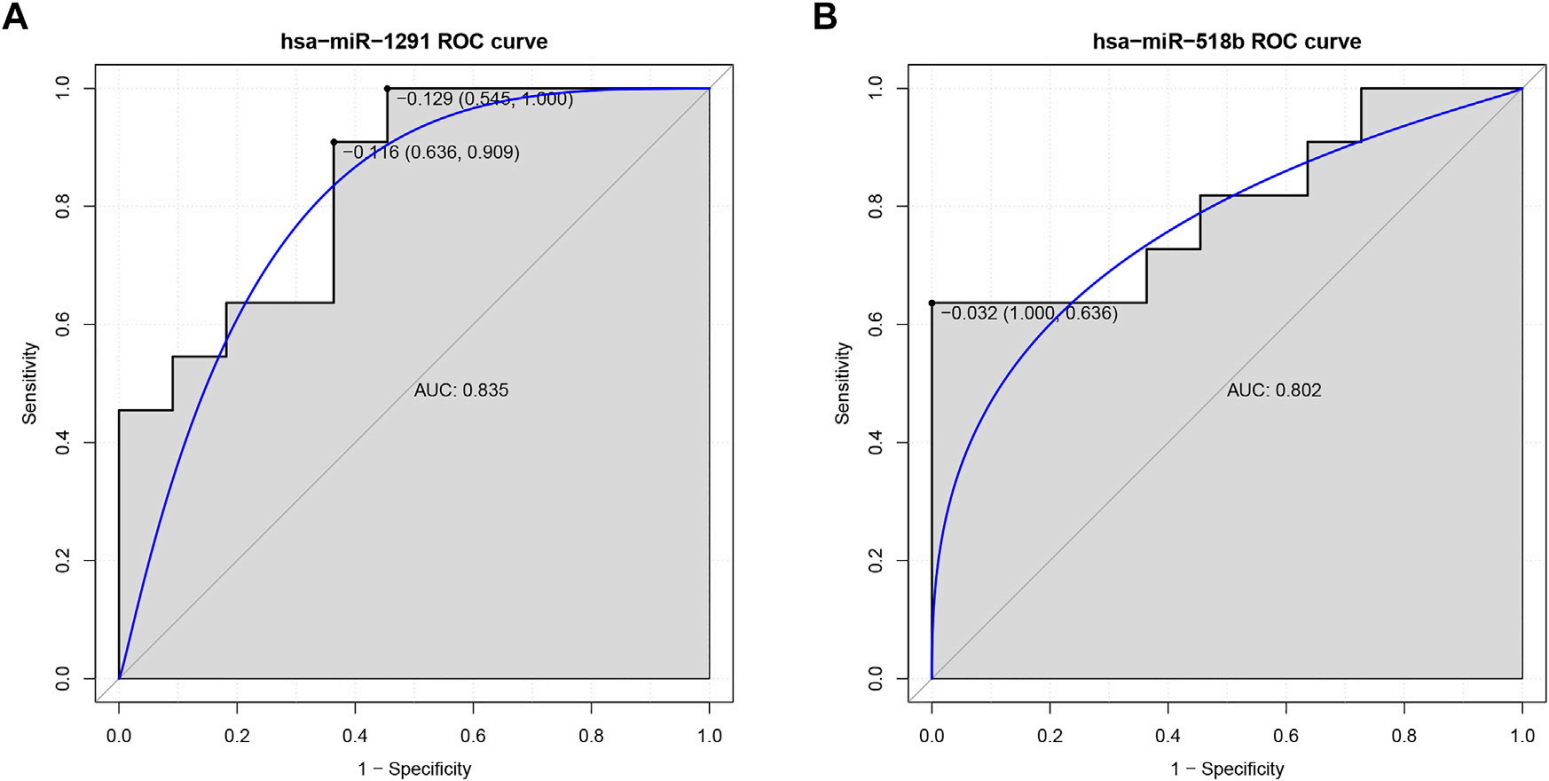
ROC analysis of up-regulated DEMis in IDD. **(A)** ROC analysis of miR-1291; **(B)** ROC analysis of miR-518b. DEMis: differentially expressed microRNAs; IDD: intervertebral disc degeneration; and ROC: receiver operating characteristic.

**FIGURE 7:**
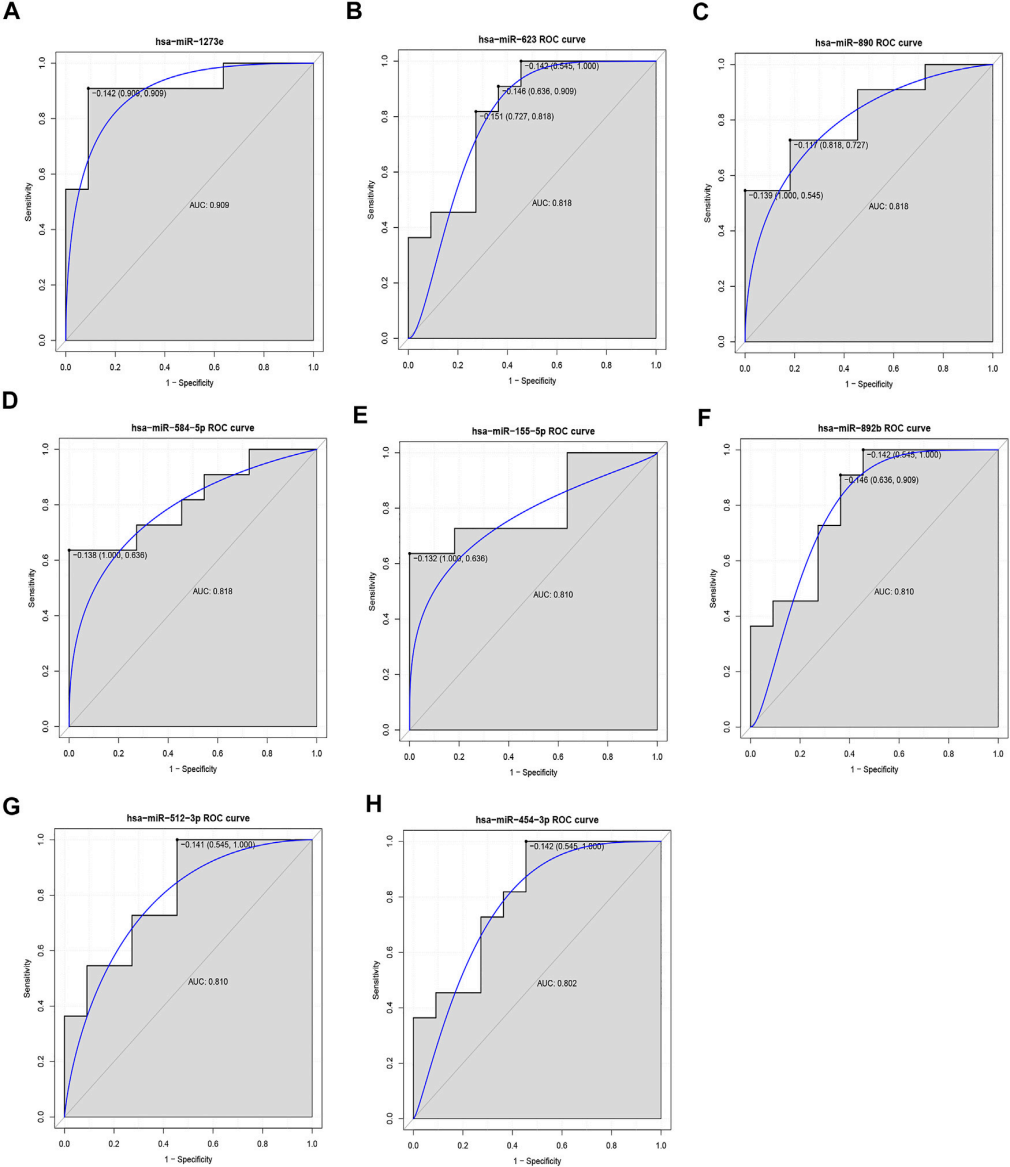
ROC analysis of down-regulated DEMis in IDD. **(A)** ROC analysis of miR-1273e, **(B)** ROC analysis of miR-623, **(C)** ROC analysis of miR-890, **(D)** ROC analysis of miR-584-5p, **(E)** ROC analysis of miR-155-5p, **(F)** ROC analysis of miR-892b, **(G)** ROC analysis of miR-512-3p, and **(H)** ROC analysis of miR-454-3p. DEMis: differentially expressed microRNAs; IDD: intervertebral disc degeneration; and ROC: receiver operating characteristic.

In our ROC analysis, the AUC of 2 out of 84 up-regulated DEMis was greater than 0.8, as shown in Figures 6A,B. The AUC of miR-1291 and miR-518b was 0.835 and 0.802, respectively. As shown in Figure 7, the AUC of 8 out of 74 down-regulated DEMis was greater than 0.8. The AUC of miR-1273e was greater than 0.9; the AUC of miR-623, miR-890, and miR-584-5p was 0.818; the AUC of miR-155-5p, miR-892b, miR-512-3p was 0.810; and the AUC of miR-454-3p was 0.802.

### Exploration of the Expression Level of DEMs and DECs

**FIGURE 8:**
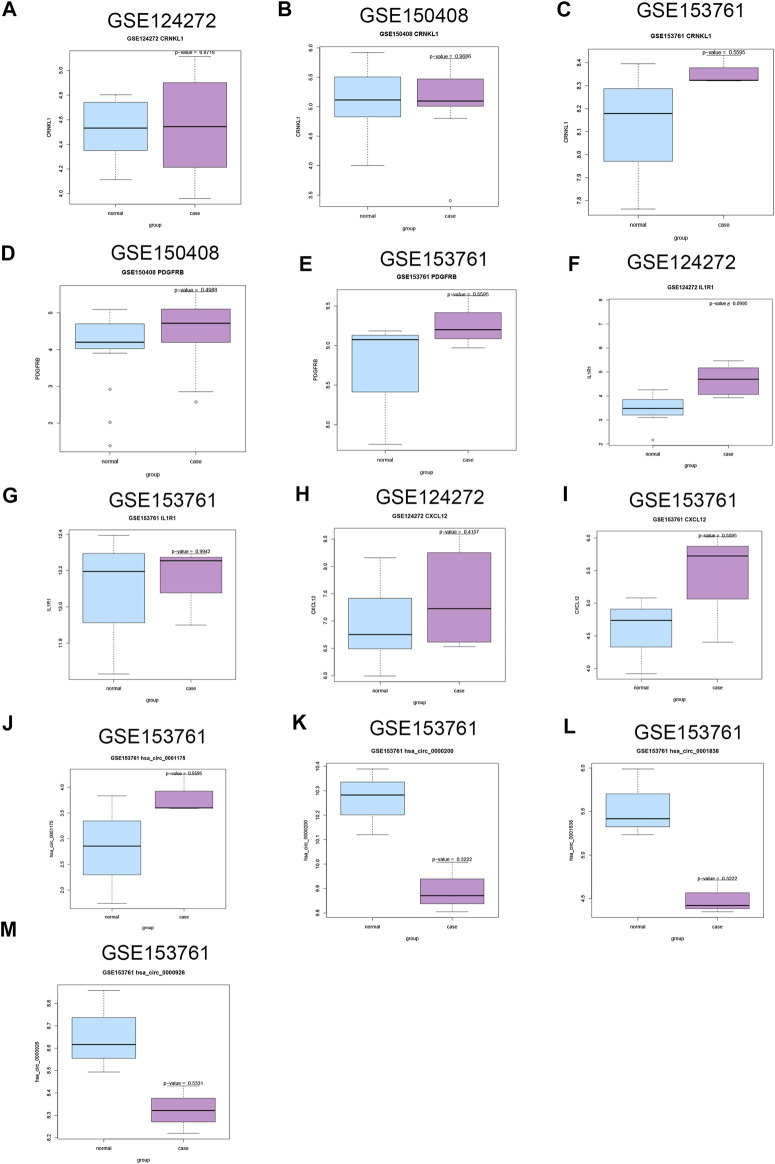
Validation of the expression of candidate DECs, DEMis, and DEMs in external GEO microarray datasets related to IDD. The difference of CRNKL1 expression between IDD and HC in GSE124272 **(A)**, GSE150408 **(B)**, and GSE153761**(C)**; the difference of PDGFRB expression between IDD and HC in GSE150408 **(D)** and GSE153761**(E)**; the difference of IL1R1 expression between IDD and HC in GSE124272 **(F)** and GSE153761 **(G)**; the difference of CXCL12 expression between IDD and HC in GSE124272 **(H)** and GSE153761 **(I)**; the difference of hsa_circ_0001175 expression between IDD and HC in GSE153761 **(J)**; the difference of hsa_circ_0000200 expression between IDD and HC in GSE153761 **(K)**; the difference of hsa_circ_0001838 expression between IDD and HC in GSE153761 **(L)**; the difference of hsa_circ_0000926 expression between IDD and HC in GSE153761 **(M)**. DEC: differentially expressed circular RNAs; DEMis: differentially expressed microRNAs: DEMs: differentially expressed mRNAs; IDD: intervertebral disc degeneration; and GEO: Gene Expression Omnibus.

For DEMs, CRNKL1 was up-regulated in IDD patients in these three datasets with no significant difference (Figures 8A–C); PDGFRB was up-regulated in IDD patients in GSE150408 (Figure 8D) and GSE153761 (Figure 8E) datasets with no significant difference; IL1R1 had a trend of significant up-regulation in IDD patients in GSE124272 (Figure 8F); and CXCL12 (Figures 8H,I) was up-regulated in both GSE124272 and GSE153761 datasets with no significant difference. For DECs, only GSE150408 stores circRNA data of IDD patients. We found that hsa_circ_0001175 was up-regulated in IDD with no significant difference; in addition, hsa_circ_0000200, hsa_circ_0000926, and hsa_circ_0001838 were down-regulated in IDD with no significant difference (Figures 8J–M).

## Discussion

In the present work, differentially expressed genes in IDD were identified and their potential roles were explored; in addition, the expression level of differentially expressed genes was validated in external IDD transcriptomic profiling datasets.

We found that CRNKL1, IL1R1, and CXCL12 were up-regulated in the discovery (GSE23130, GSE15227, GSE17077, and GSE70362) and external datasets. CRNKL1 encodes crooked neck pre-mRNA splicing factor 1, which has been reported to be a hub gene in the protein–protein interaction network identified in osteoporosis (Qian et al., 2019). It also has been identified as a potential prognostic biomarker in esophageal adenocarcinoma (Li et al., 2017). However, its expression status in IDD has not been documented. CXCL12 encodes C-X-C motif chemokine ligand 12, a stromal cell-derived alpha chemokine member of the intercrine family. Previously published articles have demonstrated that CXCL12 is implicated in IDD (Er et al., 2020; Zhong et al., 2021). Serum CXCL12 level is positively related to lumbar IDD and its clinical severity (Er et al., 2020). The miR-623/CXCL12 axis inhibits LPS-induced nucleus pulposus cell apoptosis and senescence (Zhong et al., 2021).

In this study, we found that lncRNA H19 was up-regulated in IDD. The ceRNA network suggested that up-regulated H19 might regulate the targeted effects of hsa-miR-454-3p on CRNKL1 and PDGFRB and hsa-miR-2355-5p on PBX1 and TFDP2. It is reported that H19 aggravates IDD by promoting the autophagy and apoptosis of nucleus pulposus cells through the miR-139/CXCR4/NF-kappaB axis (Sun et al., 2021). H19 could target miR-22 to modulate H_2_O_2_-induced deregulation in nucleus pulposus cell senescence, proliferation, and ECM synthesis through Wnt signaling (Wang et al., 2018). In this work, both miR-139 and miR-22 were not dysregulated in IDD. The discordant findings might be attributed to different IDD cohorts between our work and previous studies. We also found that lncRNA HOTAIR was down-regulated in IDD. The ceRNA network suggested that HOTAIR might function as a ceRNA to suppress the inhibitory effects of hsa-miR-642a-5p on MMP13, MAP4K4, and PIK3R1, thus leading to their up-regulated expression. It is reported that HOTAIR serves as a miRNA-34a-5p sponge to reduce nucleus pulposus cell apoptosis via a NOTCH1-mediated mechanism (Shao et al., 2019). In addition, HOTAIR could modulate IDD changes via the Wnt/β-catenin pathway (Zhan et al., 2019). miR-34a-5p was not dysregulated in IDD. The discordant findings might be attributed to the different IDD cohorts between our work and previous studies. Consistent with previous studies, we found that H19 and HOTAIR were dysregulated in IDD. The ceRNA mechanisms related to H19 and HOTAIR identified in this study should be validated through *in vitro* and *in vivo* studies.

It is reported that miR-623, miR-663b, miR-193a-5p, miR-376c-3p, miR-664a-5p, miR-4297, and miR-155 are significantly down-regulated in IDD and miR-2355-5p was significantly up-regulated in IDD (Wang et al., 2011; Li et al., 2015; Ji et al., 2016; Wang et al., 2016), which are in line with our analyses. It is indicated that our bioinformatics analyses were acceptable. In the present work, 10 DEMs having diagnostic value in distinguishing IDD patients from normal individuals were identified. Of those, four DEMis miR-1273e (AUC = 0.909), miR-623 (AUC = 0.818), miR-890 (AUC = 0.818), and miR-584-5p (AUC = 0.818) had high performance in distinguishing IDD from HC. Two DEMis miR-1273e and miR-623 were top 10 down-regulated DEMis in IDD. miR-890 and miR-584-5p were the top 12 and top 17 down-regulated DEMis, respectively. A series of articles have reported that miR-1273e and miR-623 play key roles in clinical disease. miR-1273e is significantly down-regulated and associated with endocapillary glomerular inflammation (Cardenas-Gonzalez et al., 2017). Inhibited miR-1273e promotes cell proliferation, invasion, and migration and inhibits cell apoptosis in gastric cancer (Dou et al., 2019). miR-623 suppresses tumor progression in hepatocellular carcinoma, gastric cancer, and pancreatic cancer (Jiang et al., 2018; Chen et al., 2019; Li et al., 2020; Ren et al., 2020). miR-890 could inhibit proliferation and invasion and induce apoptosis in triple-negative breast cancer cells by targeting CD147. miR-584-5p has been reported to be implicated in hepatocellular carcinoma, non-small-cell lung cancer, osteosarcoma, and gastric cancer. However, the biological roles of miR-1273e, miR-623, miR-890, and miR-584-5p in IDD have not been documented in the literature.

miR-518b and miR-1291 were the top 10 up-regulated DEMis in IDD, and the AUC of miR-518b and miR-1291 was 0.802 and 0.835, respectively, in the ROC analyses. miR-1291 had high connectivity with target DEMs, which targeted 96 DEMs including RASSF1 as a top 20 down-regulated DEM. Dysregulated miR-518b is involved in the progression of esophageal squamous cell carcinoma and glioblastoma (Zhang et al., 2012; Xu et al., 2017). miR-1291 has been implicated in the development of various cancers including pancreatic cancer, renal cell carcinoma, and prostate cancer (Yamasaki et al., 2013; Cai et al., 2019; Tu et al., 2019). Currently, the roles of miR-518b, miR-1291, and RASSF1 in IDD have not been investigated.

The JAK–STAT signaling pathway and cell death biological process were significant enrichment in IDD. IL-12 could aggravate IDD by stimulating TNF-alpha through the JAK–STAT signaling pathway (Chen et al., 2017). Cellular loss from cell death has been reported to contribute to the degradation of the ECM and plays an important role in the process of IDD degeneration (Zhao et al., 2006; Ding et al., 2013). In our work, the regulation of the apoptotic process and apoptotic process were significant enrichment terms. The overexpression of miRNA-143 promotes the progression of nucleus pulposus apoptosis by directly targeting BCL2 in human IDD (Zhao et al., 2017).

There are several limitations in the present work. First, the diagnostic value of 10 DEMis which had potentially diagnostic value in distinguishing IDD from HC in our analysis was not explored in a large clinical cohort. Second, the expression levels of DELs in IDD were not validated due to the fact that external lncRNA expression data generated from the IDD cohort with more than 3 IDD cases were unavailable in the GEO database at the time of article submission. Validation of the expression levels of DELs in IDD should be performed in further work. Third, the biological functions of candidate differentially expressed genes should be investigated in *in vitro* and *in vivo* studies.

## Conclusion

In summary, our study might provide an additional framework for understanding the pathogenesis of IDD and pave the way for the diagnostic and therapeutic prospective of IDD.

## Data Availability

The original contributions presented in the study are included in the article/supplementary material, further inquiries can be directed to the corresponding author.
